# Engineered carbon electrode with graphene-cyclodextrin/ferrocenyl-carnosine nanoassembly for Mn(ii) detection†

**DOI:** 10.1039/d5ra03016a

**Published:** 2025-07-08

**Authors:** Chiara Abate, Giulia Neri, Marco Abbate, Massimiliano Cordaro, Placido Giuseppe Mineo, Enza Fazio, Carmelo Corsaro, Ottavia Giuffré, Claudia Foti, Anna Piperno

**Affiliations:** a Department of Chemical, Biological, Pharmaceutical, and Environmental Sciences, University of Messina 31 Viale F. Stagno d’Alcontres 98166 Messina Italy giulia.neri@unime.it claudia.foti@unime.it; b Department of Chemical Sciences, University of Catania Catania 95125 Italy; c Department of Mathematical and Computer Sciences, Physics Science and Earth Science, University of Messina 31 Viale F. Stagno d’Alcontres 98166 Messina Italy

## Abstract

The modification of electrodes using carbon-based nanomaterials is an efficient strategy for enhancing their electrical conductivity and working surface area. In this study, we explore the ability of a screen-printed carbon electrode (SPCE) modified with graphene-cyclodextrin/ferrocenyl-carnosine (GCD/FcCAR) supramolecular assembly for the determination of Mn(ii) in an aqueous solution. Although Mn(ii) is an essential nutrient for humans, it can be toxic at elevated levels, leading to neurotoxic symptoms. Consequently, accurate analytical determination is necessary. The graphene platform (G-Alk) was derivatized with mono-6-deoxy-6-azido-β-cyclodextrin (CD) *via* a click chemistry reaction. The chemical–physical properties of the GCD system were investigated using thermogravimetric analysis, scanning electron microscopy, Raman spectroscopy, and X-ray photoelectron spectroscopy. An aliquot of GCD dispersion was cast on the surface of the SPCE (SPCE/GCD), and its electrochemical response was evaluated using cyclic voltammetry (CV) with [Fe(CN)_6_]^3−^ as the redox probe. A notable 30% increase in the signal was observed for SPCE/GCD compared with the bare SPCE. Additionally, the remarkable metal-ion complexing and electroanalytical abilities of FcCAR were leveraged in this study. Therefore, CV and differential pulse voltammetry (DPV) experiments were conducted to assess the voltammetric response of FcCAR on SPCE/GCD (SPCE/GCD/FcCAR) toward Mn(ii). DPV measurements allowed us to obtain a limit of detection (LOD) and limit of quantification (LOQ) of 0.69 and 2.3 nmol L^−1^, respectively, within a linear concentration range (0.17 ≤ [Mn(ii)]/nmol L^−1^ ≤ 48). The LOD obtained is one of the lowest reported in the literature, highlighting the potential applicability of the SPCE/GCD/FcCAR system for Mn(ii) determination in aqueous solutions.

## Abstract 2

Modifying an SPCE with graphene-cyclodextrin/ferrocenyl-carnosine supramolecular assembly improves the electrochemical detection of Mn(ii), resulting in increased sensitivity and low detection limit.

## Graphical abstract

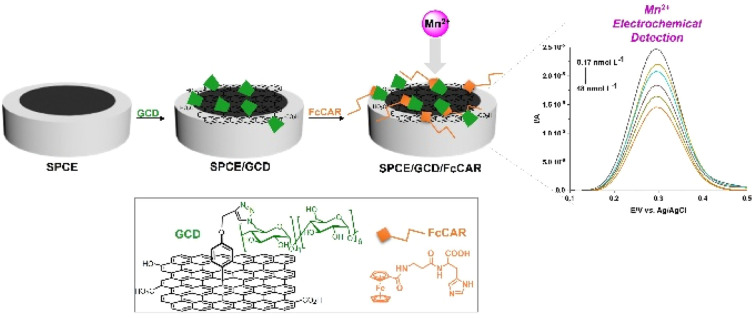

## 1 Introduction

Electrochemical sensing devices are powerful tools in analytical chemistry^1^ as they convert any measurable physical quantity into a signal that can be read, displayed, stored or controlled.^2^ Electrochemical sensors, based on screen-printed electrodes (SPEs), are particularly valued for their robustness, versatility, rapid and multiplexed analyses, cost-effectiveness, selectivity, specificity, and low detection limits.^3–5^ These features make electrochemical sensing techniques highly suitable for application across a wide range of fields,^5^ particularly for the detection of environmental contaminants (*e.g.*, metal ions, drugs, toxins, and pesticides) or pollutants in processed food (*e.g.*, phenol, sulfite, hydrazine, and hydroxylamine). Additionally, these methods offer the advantage of minimizing the use of organic solvents, reducing the number of operational steps, and streamlining the sample pretreatment process.^3,6^ Special and increasing attention has been paid to the design of electrochemical sensors for the accurate and sensitive detection of analytes. In this regard, electrode surface modification can help overcome the high redox potential and low sensitivity of electroactive materials on unmodified electrodes, accelerating electrode kinetics and enhancing sensitivity in the detection process.^6–10^ These features are most effectively achieved using the drop-casting method, which avoids the coffee-ring effect.^5^ The employment of carbon-based nanomaterials (CBNs) as surface modifiers offers several advantages, including (i) an increased active surface area, (ii) enhanced charge transfer due to high electrical conductivity, and (iii) the ability to graft specific recognition elements owing to their versatile surface chemistry, providing easy derivatization.^5,11^

In the last years, it has been demonstrated that the derivatization of CBNs with β-cyclodextrins (βCDs) results in nanohybrid systems, in which the synergistic interaction between the constituent building blocks optimally combines their individual properties.^12–15^

Our recent work involved the development of modified screen-printed carbon electrodes (SPCEs) using a nanoassembled carbon nanotube-cationic cyclodextrin (CNT-CDs) and ferrocenyl-carnosine (FcCAR) system for Hg(ii) sensing.^16^ It combines the chelating properties of the FcCAR ligand toward Hg(ii)^17^ with the ability of carbon nanomaterials to improve electrical conductivity and expand the electrode surface area through structural modifications.^16^ Graphene and its derivatives also improve electrochemical performance, and therefore, they are used in sensing and biosensing applications.^18–20^

**Fig. 1:**
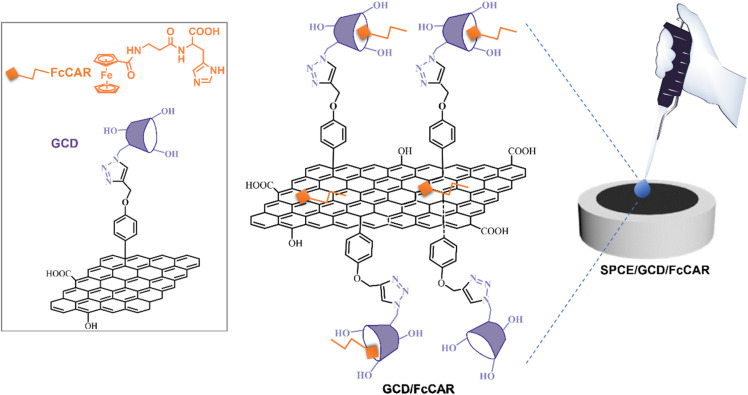
Representative illustration of SPCEs modified with the nanoassembled GCD/FcCAR system. The chemical structures of GCD and FcCAR are shown on the left.

In the present study, we exploit the peculiar properties of the FcCAR unit supramolecularly assembled with a graphene-β cyclodextrin (GCD) nanoplatform (Fig. 1). GCD offers high loading capacity owing to its larger surface area and excellent electrical conductivity;^21^ however, its hydrophobic nature and tendency to form irreversible agglomerates in water limit its applications. Therefore, proper functionalization of graphene systems with suitable compounds, such as cyclodextrins (CDs), is pivotal to overcome these limitations.^12,22^

In this context, the graphene platform (G-Alk) was covalently bonded to CD, a strategy that prevents gradual CD detachment, a common issue when CDs are not covalently attached.^23^ Moreover, the use of a click chemistry reaction to synthesize the GCD system is a relatively unexplored approach for preparing hybrid materials within the field of electrochemical sensing.^23^ Covalent conjugation with CDs further introduces new binding sites through their hydrophilic outer rims and hydrophobic inner cavities, facilitating supramolecular complexation and the formation of a stable host–guest inclusion complex involving the GCD platform and FcCAR unit (GCD/FcCAR).^16^

The chemical composition and morphology of the novel GCD system were characterized *via* thermogravimetric analysis (TGA), scanning electron microscopy (SEM), X-ray photoelectron spectroscopy (XPS), and Raman spectroscopy. An update on the synthesis of FcCAR, as previously reported in our recent publication,^17^ is also provided. Furthermore, the formation of a supramolecular complex between GCD and FcCAR on the SPCE was confirmed through XPS analysis.

The electrochemical response of the new supramolecular GCD/FcCAR assembly was evaluated through voltammetric detection of Mn(ii), by means of cyclic voltammetry (CV) and differential pulse voltammetry (DPV).

Manganese (Mn) is an essential element for living systems, including humans, and it is absorbed through food and water.^24^ However, elevated levels of Mn can lead to neurotoxic symptoms and contribute to neurodegeneration and developmental effects.^25^ Although Mn exists in various oxidation states in many natural water sources, Mn(ii) is one of the most significant forms in terms of environmental and biological relevance. Therefore, accurate and sensitive detection of trace levels of Mn(ii) is crucial.^26,27^

To this end, a novel electrochemical approach has been proposed that exploits the response of FcCAR on SPCE/GCD (SPCE/GCD/FcCAR) toward Mn(ii), with the aim of developing a simple and highly sensitive method for Mn(ii) detection. The new system demonstrated promising analytical capabilities for detecting Mn(ii) in aqueous solutions with limit of detection (LOD) and limit of quantification (LOQ) values of 0.69 and 2.3 nmol L^−1^, respectively, in the concentration range 0.17 ≤ [Mn(ii)]/nmol L^−1^ ≤ 48. This study lays the basis for the development of an analytical procedure useful for the determination of Mn(ii) in real natural waters.

## 2 Results and discussion

### 2.1 Synthesis and physicochemical characterization of the GCD nanoplatform

Chemical and structural modifications of carbon-based materials can significantly enhance their processability and sensing performance.^16^

**Fig. 2:**
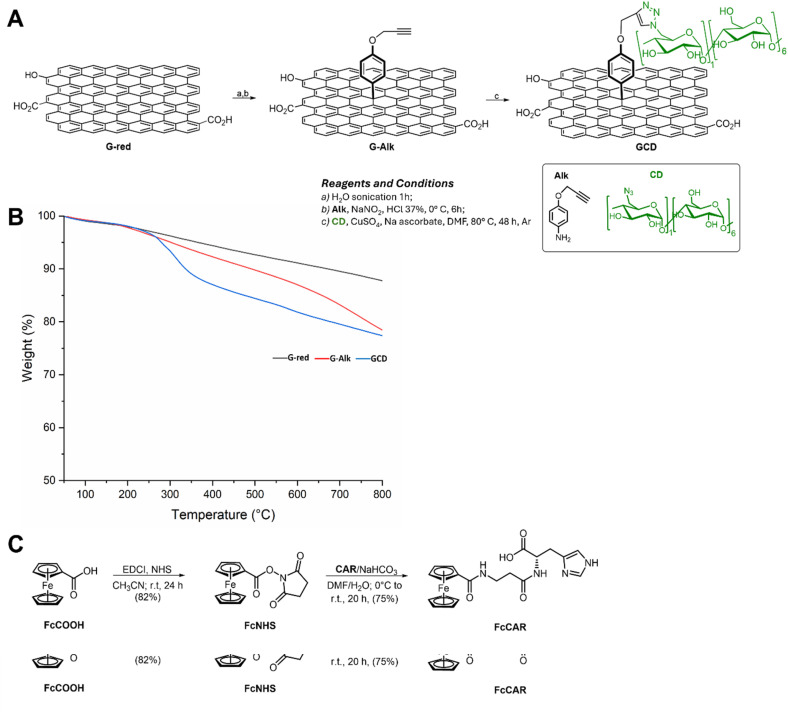
Schematic representation of the (A) GCD and (C) FcCAR synthesis protocols. (B) TGA profiles of G-red (black line), G-Alk (red line) and GCD (blue line) under nitrogen flow.

A GCD nanoplatform with high affinity for FcCAR was synthesized from commercial graphene oxide (GO) using a three-step synthetic procedure (Fig. 2A). GO was chemically reduced to G-red, partially restoring its sp^2^ network, improving the electrical properties of graphene, and making it more suitable for electrochemical sensing applications. Subsequently, G-red was derivatized with an alkyne-terminated group (G-Alk),^28^ creating anchorage sites for CD grafting. The GCD nanosystem was obtained using a click chemistry reaction between the alkyne moiety on the G surface and the azide groups of mono-6-deoxy-6-azido-β-cyclodextrin (CD).^29^ Unambiguous evidence of the functionalization of the graphene platform was provided *via* TGA analysis (Fig. 2B). G-red exhibited significantly higher thermal stability, with a constant mass loss up to 800 °C, while G-Alk displayed an initial degradation point at 180 °C, followed by continuous degradation till 800 °C (Fig. 2B). As per the TGA thermogram, the amount of the alkyne moiety grafted on the graphene surface was found to be 0.37 mmol g^−1^ (≈5% weight loss, with respect to G-red residue at 600 °C). In agreement with literature data,^12,30^ the TGA profile of graphene decorated with CD shows a different thermal profile compared with the precursors (G-red and G-Alk), with two stages of weight loss: from 100 °C to 210 °C, due to the loss of residual water, and from 250 °C to 600 °C, due to the loss of CD units (Fig. 2B). The amount of CD grafted on GCD was determined to be 44 μmol g^−1^ (≈5% weight loss, with respect to G-Alk residue at 600 °C).

The synthesis of FcCAR (Fig. 2C) was optimized compared with previously reported procedures,^17^ resulting in the higher yield and purity of the final product. The use of EDCI and NHS as coupling reagents enabled the preparation of the Fc-NHS derivative without the formation of a dicyclohexylurea byproduct. After purification using a chromatography column, the intermediate was coupled with CAR, and the crude reaction mixture was further purified *via* chromatography using a gradient of DCM to DCM : MeOH (1 : 1) as the eluent. Purification under weak acidic conditions, due to the silica support, prevented the formation of ferrocenyl aggregates, which are typically observed when water is used in the synthesis and purification process.

FcCAR was fully characterized using NMR spectroscopy. The proton signals of the CAR and Fc moieties were clearly distinguished in ^1^H-NMR spectra. The chemical shifts of protons in the CAR moiety matched those of l-carnosine,^31^ under identical experimental conditions (D_2_O as the deuterated solvent and neutral pH) (Fig. S1 and S2†). An evident downfield shift of H-11 protons adjacent to nitrogen was observed because of the formation of amide. The enantiotopic protons at C-6 were distinguished in two distinct peaks at 2.84 and 3.02 ppm, while the H-7 proton signal appeared as a double doublet at 4.33 ppm. Other protons showed no notable differences, except for the imidazole protons, which exhibited large variations in chemical shift, depending on sample concentration and pH. Fc protons were anisotropic on the derivatized cyclopentadienyl ring, and two different signals were observed at 4.61 ppm and 4.40 ppm for H15, 18 and H16, 17, respectively. The five protons on the other cyclopentadienyl ring were equivalent and appeared as a single signal at 4.09 ppm. The ^13^C NMR spectrum of FcCAR clearly showed three peaks for the carbonyl carbons at low fields (173–177 ppm), corresponding to two amides and one carboxylic acid group.

**Fig. 3:**
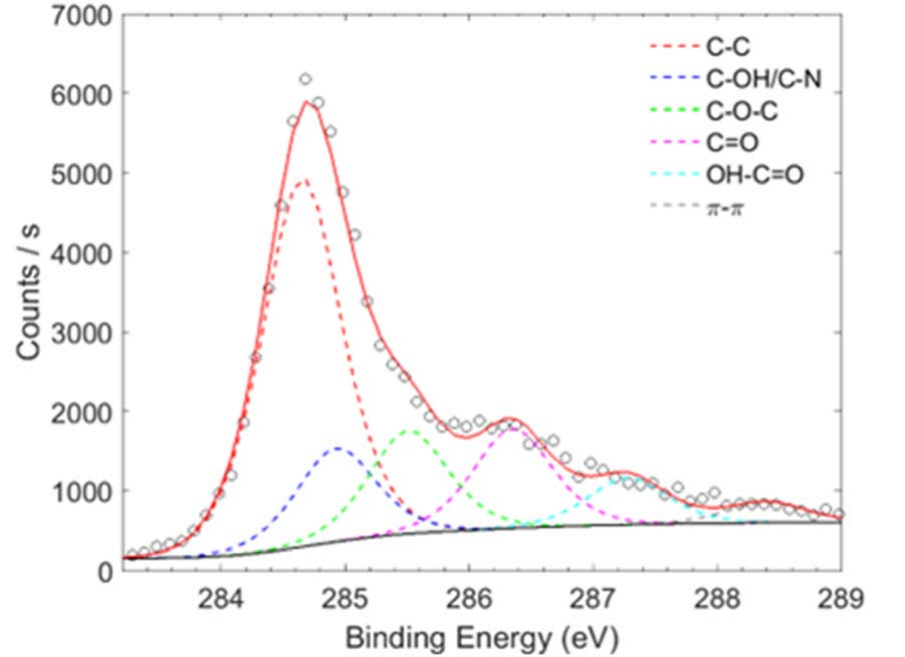
XPS C 1s high resolution spectra of the GCD sample deconvoluted using six Gauss–Lorentzian functions.

Surface composition is a critical factor influencing the electrochemical sensitivity of carbon-based nanomaterials. Therefore, the elemental composition of the obtained samples (Table 1) was investigated using X-ray photoelectron spectroscopy (XPS) survey spectra (not shown). The slight increase in oxygen and nitrogen species in GCD confirms the successful derivatization of graphene with CD units, compared with G-Alk, as indicated by the increase in surface carbon oxidation and C–N bonds (Table 1 and Fig. 3). Moreover, a high degree of compositional uniformity in the GCD nanosystem was observed by evaluating atomic species at different points on the surface, confirming the efficacy of washing cycles in removing unbonded material from the platform surface.

**Table 1:** Surface elemental composition estimated by wide scan spectra and the percentage of carbon bonding configurations estimated by convolving the HR C 1s spectra

Sample	C (%)	O (%)	N (%)	S (%)	C–C (%)	C–OH/C–N (%)	C–O–C (%)	C=O (%)	OH–C=O (%)	π–π (%)
G-Alk	85.4	11.1	3.5	—	56.1	12.6	16.1	9.0	4.7	1.5
GCD (#1)	82.0	13.7	4.3	—	50.3	12.8	14.1	13.6	6.4	2.8
GCD (#2)	80.8	14.3	4.9	—	52.1	13.3	13.7	13.9	5.1	1.9

**Fig. 4:**
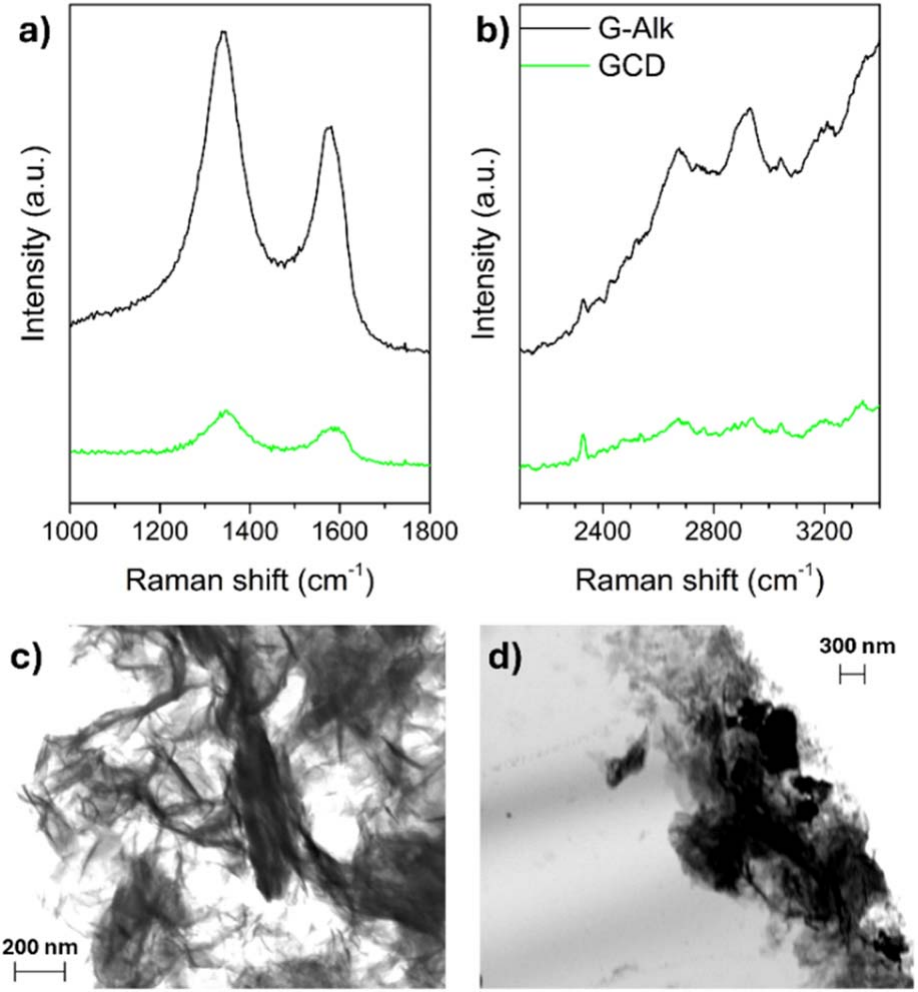
Raman spectra of G-Alk and GCD investigated samples (a and b). SEM images of G-Alk (c) and GCD (d).

The derivatization of G-Alk with CDs was confirmed using Raman spectroscopy. The Raman spectra of G-Alk and GCD (Fig. 4a) show the typical bands of carbon-based materials, namely, the G and D bands centered at about 1660 cm^−1^ and 1380 cm^−1^, respectively. The broadening and shift of these bands toward a higher wavenumber are due to graphene plane functionalization (GCD), which induces a size reduction in-plane sp^2^ domains. Moreover, the derivatization of the graphene surface is proved by D and G band intensity decrease (Fig. 4a), together with the disappearance of the G* band and the second-order overtone 2D mode in the 2400–2700 cm^−1^ range (Fig. 4b).^32,33^

The SEM images of G-Alk (Fig. 4c) and GCD (Fig. 4d) show well-defined edges and many thin layers of graphene. Particularly, homogeneous and quite smooth overlapped sheets are evident in the G-Alk sample, whereas GCD exhibits amorphous aggregates on the layer surface due to the functionalization with CDs.

**Fig. 5:**
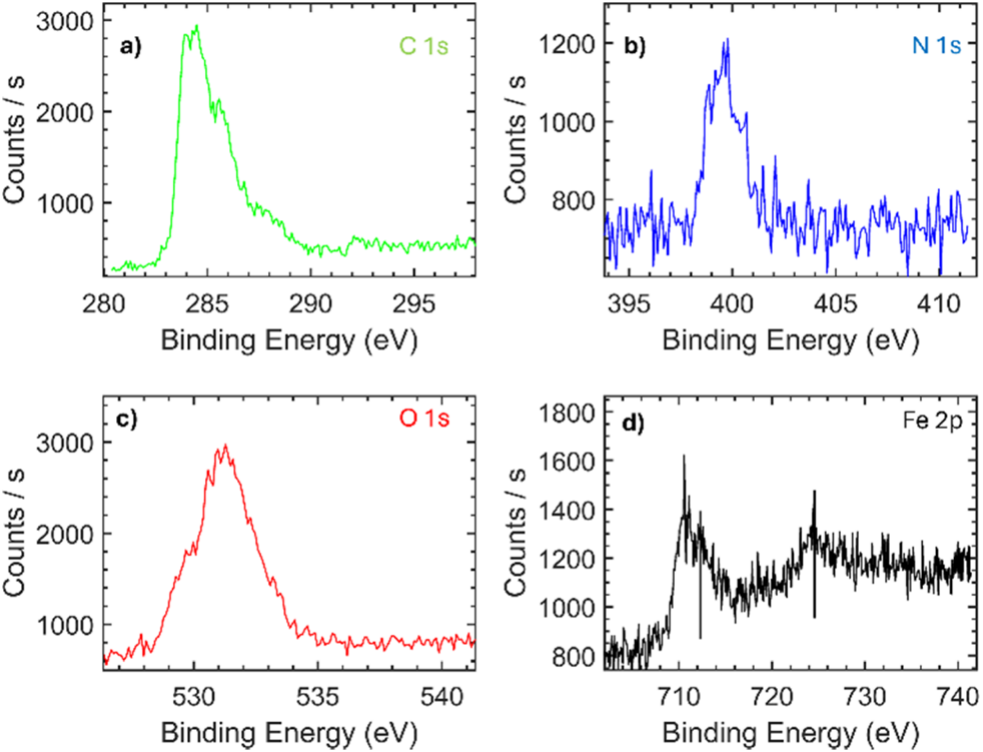
XPS C 1s, N 1s, O 1s, and Fe 2p high resolution spectra of the SPCE/GCD/FcCAR sample.

To assess the formation of a supramolecular complex between the GCD system and FcCAR unit, XPS analysis was performed on the bare SPCE, SPCE modified with GCD (SPCE/GCD), and SPCE modified with GCD and FcCAR (SPCE/GCD/FcCAR). The evaluation of 4.0% Fe 2p amount in SPCE/GCD/FcCAR, which is related to the ferrocenyl fragment, supports the formation of the GCD-FcCAR complex. Particularly, the bands centered at 712.5 eV (Fe 2p_1/2_) and 724.5 eV (Fe 2p_3/2_) in SPCE/GCD/FcCAR are ascribed to Fc^34,35^ (Fig. 5). Additionally, the increase in N 1s and O 1s percentages (Table 2) from 4.4% and 11.3% in SPCE/GCD to 9.5% and 24.0% in SPCE/GCD/FcCAR, respectively, reflects the presence of the carnosine moiety, further confirming the derivatization of GCD with FcCAR.

**Table 2:** Surface elemental compositions of the different electrodes, SPCE, SPCE/GCD, and SPCE/GCD/FcCAR, as estimated by wide scan spectra

Electrodes	C (%)	O (%)	N (%)	Fe (%)
SPCE	85.8	14.2	—	—
SPCE/GCD	84.3	11.3	4.4	—
SPCE/GCD/FcCAR	62.5	24.0	9.5	4.0

### 2.2 Electrochemical investigation

**Fig. 6:**
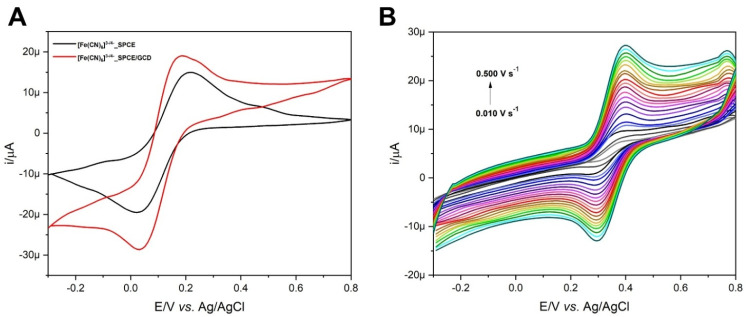
(A) CVs of [Fe(CN)_6_]^3−^ (1 mmol L^−1^) in KCl (0.1 mol L^−1^) on bare SPCE (black line) and SPCE/GCD (red line). (B) CVs of FcCAR (0.5 mmol L^−1^) in KCl (0.1 mol L^−1^) *vs.* the scan rate (*ν*/V s^−1^) on SPCE/GCD.

Here, 1 μL of GCD dispersion in DMF (3 mg mL^−1^) was cast on the working electrode (WE) surface, and the modified SPCE (SPCE/GCD) was dried under a DMF atmosphere (80 °C). Then, the electrochemical response was evaluated through cyclic voltammetry (CV) using [Fe(CN)_6_]^3−^ as the redox probe. Fig. 6A shows ∼30% amplification of the electrochemical signal by switching from the SPCE to SPCE/GCD. The enhanced electron transfer between potassium ferricyanide and the WE surface is due to the electrical conductivity of the GCD material, which demonstrates the deposition of the material on the electrode surface. However, it did not support further addition of the conductive material, indicating saturation. Voltammetric signals, in terms of anodic (*E*_a_) and cathodic (*E*_c_) potentials (*vs.* Ag/AgCl) and anodic (*i*_a_) and cathodic (*i*_c_) peak currents are as follows: for the SPCE, *E*_a_ = 0.216 V (*i*_a_ = 15.21 μA) and *E*_c_ = 0.021 V (*i*_c_ = −19.68 μA); for SPCE/GCD, *E*_a_ = 0.188 V (*i*_a_ = 19.19 μA) and *E*_c_ = 0.030 V (*i*_c_ = −28.73 μA). The slight shift in potential values (Δ*E*_a_ = 0.028 V and Δ*E*_c_ = 0.009 V) could be attributed to attractive interactions between the anionic [Fe(CN)_6_]^3−^ and functional groups on the electrode surface of SPCE/GCD.

The electrochemical behavior of FcCAR (0.5 mmol L^−1^) in KCl (0.1 mol L^−1^) on SPCE/GCD at 0.100 V s^−1^ was investigated and compared with that obtained on the bare SPCE. Fig. S3† shows the cyclic voltammograms of FcCAR on the SPCE and SPCE/GCD with a one-electron reversible redox process, similar to the one occurred on SPCE/CNT-CD.^16^ For SPCE/GCD, the *E*_a_ and *E*_c_ values are 0.395 V and 0.286 V (*vs.* Ag/AgCl), respectively. Comparing these potential values with those obtained on bare SPCEs, only *E*_c_ undergoes slight cathodic shift as a result of functionalization on the WE surface.

The electrochemical behavior of FcCAR was also studied at various scan rates (0.01 ≤ V s^−1^ ≤ 0.500) on SPCE/GCD (Fig. 6B). The relationship between the peak current (i) and scan rate (*ν*) provides information on whether the electrochemical process is controlled by adsorption or diffusion.^36^ Particularly, the *i*_a_ and *i*_c_ peak currents vary linearly with *ν* (Fig. S4,†eqn (1) and (2)), indicating that the redox process is mainly controlled by adsorption:^4,37^

1 *i*_a_ (μA) = 4.031*ν* (V s^−1^) + 8.081, *R*^2^ = 0.993

2 *i*_c_ (μA) = −3.141*ν* (V s^−1^) + 2.452, *R*^2^ = 0.994

First, we analyzed the electrochemical behavior of SPCE/GCD/FcCAR. The voltammetric response of FcCAR in KCl (0.1 mol L^−1^) in the presence of Mn(ii) (0.17 ≤ nmol L^−1^ ≤ 417) was evaluated *via* CV (at 0.100 V s^−1^, Fig. S5†) and differential pulse voltammetry (DPV). While the potential values do not change in the presence of Mn(ii), confirming that the Fc/Fc^+^ redox couple is the moiety responsible for the redox process, *i*_a_ peak currents linearly decrease as the metal concentration increases. To highlight that the decrease in current is mediated by the ligand (FcCAR), in the same Fig. S5,† the blank signal, *i.e.*, the CV of SPCE/GCD/Mn, was also reported.

**Fig. 7:**
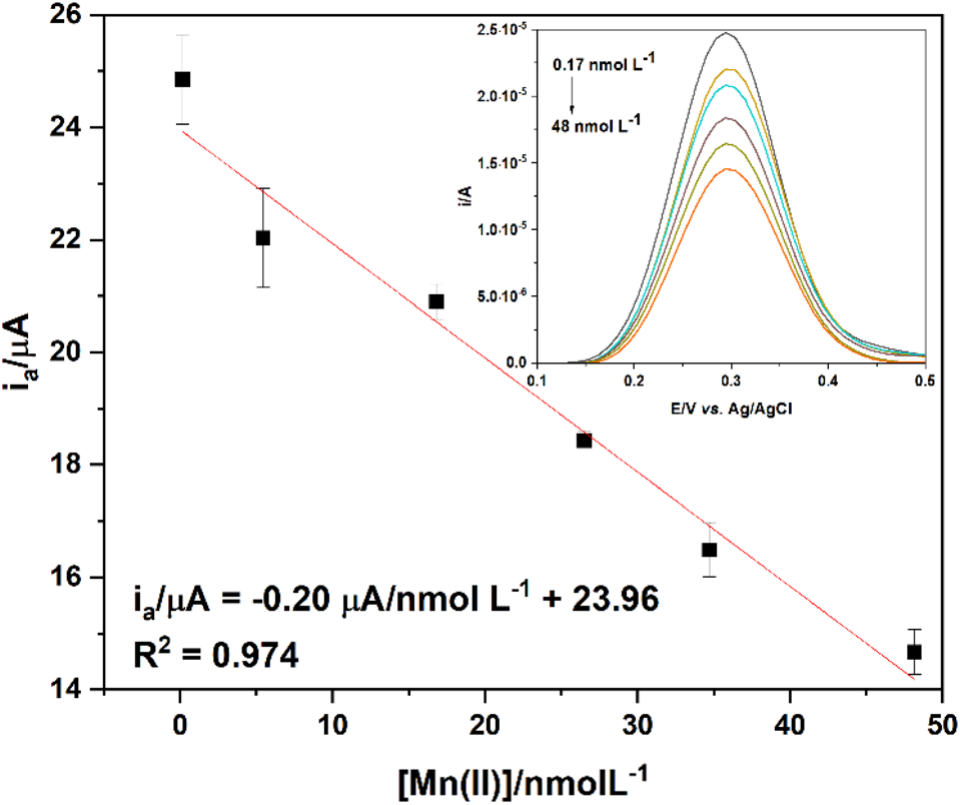
Linear range of the anodic (*i*_a_) peak currents of FcCAR *vs.* Mn(ii) concentration (0.17 ≤ [Mn(ii)]/nmol L^−1^ ≤ 48) on SPCE/GCD. The inset shows the respective DPV scans.

The *i*_a_ values obtained through DPV were plotted *vs.* Mn(ii) concentration, and the linear concentration range (0.17 ≤ nmol L^−1^ ≤ 48) is shown in Fig. 7. The limit of detection (LOD) and limit of quantification (LOQ) were determined using the 3*σ* s^−1^ and 10*σ* s^−1^ approaches,^38^ where *σ* is the standard of seven independent measurements of a blank sample, and *s* is the slope of the calibration plot (Fig. 7). The LOD and LOQ values were found to be 0.69 nmol L^−1^ and 2.3 nmol L^−1^, respectively, with a sensitivity of 0.20 μA nmol^−1^ L^−1^. Compared with other LOD values reported in the literature^39–43^ (Table 3), this system (SPCE/GCD/FcCAR) shows high performance in detecting Mn(ii) in aqueous solutions. Reproducibility was 5.6%, expressed as relative standard deviation (RSD%) of three sets, each consisting of three consecutive DPV scans, performed on different SPCE/GCD on solutions containing FcCAR (0.5 mmol L^−1^) and Mn(ii) (2.3 nmol L^−1^). Under the same conditions, accuracy was 94%, expressed as 100% − error rate (*i.e.*, (average actual value − average predicted value)/average actual value × 100). The voltammetric response of SPCE/GCD/FcCAR remains almost constant for six months, highlighting the good storage stability and robustness of the drop-casting method used.

**Table 3:** Comparison with the literature data (type of electrode, linear range and LOD) reported over the past 10 years

Electrode	Linear range	LOD (nmol L^−1^)	Ref.
SPCE/GCD/FcCAR	0.17–48 nmol L^−1^	0.69	This work
Pd	455 nmol L^−1^–10.9 μmol L^−1^	334.00	44
Pyrolytic graphite	12.3 nmol L^−1^–0.1 mol L^−1^	4.78	45
Pt	91–910 nmol L^−1^	16.30	46
Au	0.11–0.32 μmol L^−1^	31.00	47
Additive-manufactured electrode	9.1 nmol L^−1^–2.7 μmol L^−1^	1.60	48
GC	2.5–200 μg L^−1^	9.64	49
GC	1–7 μmol L^−1^	430.00	50
Pt	91 nmol L^−1^–1.82 μmol L^−1^	10.10	51
SPE/Au NPs/rGO/Fe_3_O_4_ NPs/l-cysteine	0.5–300 μg L^−1^, 62–1500 μg L^−1^	520.00, 23 707.56	52
IIP/MWCNT-Chit-IL/GCE edge-plane pyrolytic graphite electrode	2–9 μmol L^−1^	150.00	53
Indium tin oxide	5–500 μg L^−1^	7.65	54
Gold electrodes	—	4640.00	55

## 3 Experimental

### 3.1 Materials and reagents

Potassium ferricyanide(iii) (K_3_Fe(CN)_6_, ≥99%), potassium chloride (KCl, extra pure), manganese(ii) chloride tetrahydrate (MnCl_2_·4H_2_O, ≥99%), hydrazine (N_2_H_4_), 4-nitrophenol, propargyl bromide solution, potassium carbonate (K_2_CO_3_), tin(ii) chloride (SnCl_2_), sodium hydroxide (NaOH), sodium nitrite (NaNO_2_), hydrochloric acid 37% (HCl), copper sulfate (CuSO_4_), sodium ascorbate, ferrocenecarboxylic acid, *N*-hydroxysuccinimide (NHS), 1-ethyl-3-(3-dimethylaminopropyl)carbodiimide (EDCI), silica, carnosine (CAR), sodium bicarbonate (NaHCO_3_) and other solvents employed were purchased from Sigma Aldrich. Graphene oxide was purchased from Graphenea. Mn(ii) solutions were prepared by weighing and dissolving the respective salt in MOPS buffer containing 30 mmol L^−1^ 3-(*N*-morpholino) propanesulfonic acid, 10 mmol L^−1^ sodium acetate, and 1 mmol L^−1^ ethylenediaminetetraacetic acid disodium salt (EDTA) and adjusting the pH to 7. These products were also purchased from Sigma Aldrich.

### 3.2 Instruments and characterizations

TGA was performed using a PerkinElmer TGA7 instrument with Pyris software (PerkinElmer) for data acquisition and analysis. Briefly, approximately 5 mg of each sample was placed in a platinum pan and kept at 50 °C in a nitrogen atmosphere (flow rate of 60 mL min^−1^) until thermal equilibrium was reached. The sample was then heated at a scan rate of 10 °C min^−1^ from 50 to 800 °C. A baseline, recorded under the same experimental conditions with an empty platinum pan, was subtracted from each thermogram prior to data analysis. The amount of the organic material loaded onto G-red was evaluated considering the weight loss of GCD and of the parent compounds (G-red and G-Alk) at 600 °C.

XPS spectra were acquired using the K-alpha system of Thermo Scientific, equipped with a monochromatic Al-Kα source (1486.6 eV), operating in the constant analyser energy (CAE) mode with a pass energy of 20 eV for high-resolution spectra and a spot size of 400 μm. The Avantage software-equipped K-alpha system was used to fit every core-level photoemission peak: Gauss–Lorentzian shape functions and a Shirley background were used to reproduce the experimental data.

Raman spectra were recorded with the XploRA (Horiba) spectrometer in the range 1000–2700 cm^−1^ using a diode laser (*λ* = 532 nm) as an excitation source integrated for 50 s and collected with a charge-coupled detector (CCD) using a microscope objective with a 50× focal length.

The morphology of the samples was examined by adding the suspensions dropwise onto a holey copper grid. Then, a drying step was performed in ambient air at room temperature. SEM micrographs were recorded at an accelerating voltage of 30 kV using a Zeiss Gemini 2 scanning electron microscope.

Freeze-drying was carried out using a Labconco FreeZone lyophilizer.

NMR spectra (^1^H, ^13^C) were recorded on a Varian 500 MHz spectrometer at 25 °C.

### 3.3 Synthesis of GCD

Alkyne-terminated graphene (G-Alk) was synthetized as reported in a previous study,^28^ and the amount of alkyne group grafted onto the graphene platform was determined *via* TGA to be ≈5 wt%, corresponding to ≈0.37 mmol g^−1^. Mono-6-deoxy-6-azido-β-cyclodextrin (CD) was obtained following the method described in a published study.^29^

G-Alk (270 mg, 0.175 mmol of the alkyne moiety) was dispersed in DMF (15 mL) *via* sonication. Then, CD (229.3 mg, 0.175 mmol), CuSO_4_ (28 mg, 0.175 mmol), and sodium ascorbate (45 mg, 0.227 mmol) were added, and the reaction was refluxed at 80 °C for 48 h under argon flow (Fig. 2A). The mixture was cooled to room temperature, diluted with Milli-Q-water (MqW), and centrifuged at 4500 rpm for 25 min. The precipitate was collected and resuspended in a MqW : MeOH (2 : 1) mixture and sonicated for 20 min, followed by an additional centrifugation step at 4500 rpm for 35 min. The washing step was repeated one more time. GCD was lyophilized, and 288 mg of the product was obtained. TGA analyses showed that the functionalization degree of GCD was approximately 5%, corresponding to 44 μmol g^−1^ of CD on G-red.

### 3.4 Synthesis of FcCAR

Ferrocenecarboxylic acid (1 g; 4.34 mmol), *N*-hydroxysuccinimide (NHS, 500 mg; 4.34 mmol), and 1-ethyl-3-(3-dimethylaminopropyl)carbodiimide (EDCI, 850 mg; 5.47 mmol) were dissolved in anhydrous acetonitrile (5 mL) under an argon atmosphere. The solution was stirred at room temperature overnight. The solvent was removed *via* rotary evaporation, and the resulting mixture was purified *via* column chromatography using SiO_2_ as the stationary phase and dichloromethane (DCM) as the eluent, yielding 2,5-dioxopyrrolidin-1-yl ferrocenoate (1.16 g; 3.54 mmol; 82% yield). All spectroscopic data were consistent with those previously reported.^56^

A solution of ferrocenyl ester derivative (530 mg; 1.63 mmol) dissolved in dimethylformamide (DMF, 10 mL) was slowly added to a cold solution of carnosine (407 mg; 1.79 mmol) and sodium bicarbonate (NaHCO_3_, 126 mg; 1.49 mmol) in a 1 : 1 mixture of H_2_O/DMF (50 mL) under stirring. The reaction mixture was stirred at room temperature for 20 h. The crude product was purified through silica gel column chromatography using a gradient elution with DCM to DCM:methanol (MeOH, 1 : 1), resulting in the formation of ferrocenyle-carnosine (FcCAR) (536 mg, 75% yield) (Fig. 2C).

^1^H- and ^13^C-NMR analyses of FcCAR (Fig. S1 and S2†) were performed in D_2_O on a Varian 500 MHz spectrometer.

^1^H-NMR (D_2_O, 500 MHz): *δ* 7.70 (s, 1H, H-2), 6.85 (s, 1H, H-4), 4.62–4.61 (m, 2H, H-15,18) 4.40 (s, 2H, H-16,17), 4.33 (dd, 1H, *J* = 4.6 Hz, *J* = 8.6 Hz, H-7), 4.09 (s, 5H, H-19-23), 3.39 (t, 2H, *J* = 6.3 Hz, H-11), 3.02 (dd, 1H, *J* = 15.4 Hz, *J* = 4.6 Hz, H-6), 2.84 (dd, 1H, *J* = 15.4 Hz, *J* = 8.6 Hz, H-6′), 2.45–2.41 (m, 2H, H-10).

^13^C-NMR (D_2_O, 125 MHz): *δ* 177.1, 173.8, 173.2, 134.3, 131.3, 116.8, 73.6, 71.5, 71.4, 70.0, 68.3, 68.2, 54.6, 48.8, 35.7, 35.2, 28.1.

### 3.5 Voltammetric equipment and measurements

All electrochemical measurements were performed in KCl (0.1 mol L^−1^) aqueous solutions using a PC-controlled electrochemical workstation with a three-electrode cell configuration (Metrohm DropSens screen-printed electrodes (SPEs), DRP-DSC4MM 72098), connected to a μAutolab potentiostat-galvanostat type III (EcoChemie) with an IME663 interface. Screen-printed carbon electrodes (SPCEs), consisting of carbon working and counter electrodes as well as Ag/AgCl reference electrodes, were used. The WE diameter and geometric area are 4 mm and 0.11 cm^2^, respectively. The electrode surface was modified through drop-casting 1 μL of a homogeneous dispersion of GCD in DMF (3 mg mL^−1^), obtained through probe sonication treatment (5 min, 50% power), and drying the modified SPCEs (SPCE/GCD) at 80 °C under a DMF atmosphere (for at least 1 h). Voltammograms were processed using General Purpose Electrochemical System (GPES) software, version 4.9 (EcoChemie B.V.). The voltammetric response of SPCE/GCD was assessed *via* CV using [Fe(CN)_6_]^3−^ (1 mmol L^−1^) in KCl solution (0.1 mol L^−1^) and was performed from −0.3 V to +0.8 V (*vs.* Ag/AgCl) at 0.1 V s^−1^. The electrochemical behavior of FcCAR (0.5 mmol L^−1^) in KCl solution (0.1 mol L^−1^) was investigated at different scan rate values (0.010 ≤ V s^−1^≤ 0.500). The electrochemical activity of FcCAR was also studied in the presence of Mn(ii) (0.17 ≤ nmol L^−1^ ≤ 417) using CV, performed at 0.100 V s^−1^ with a step potential of 0.010 V, and differential pulse voltammetry (DPV) with a potential step and amplitude of 0.010 V and 0.1 V, respectively. CV and DPV were carried out in the following potential range: −0.3 ≤ E/V *vs.* Ag/AgCl ≤ 0.8.

## 4 Conclusions

In the present work, we investigated the properties of a novel nanoassembled GCD/FcCAR system for the electrochemical detection of Mn(ii). The adopted synthetic strategy for FcCAR gave a product with better purity compared with the one previously reported in the literature.^17^ GCD was synthesized *via* a multistep process involving the reduction of commercial GO, followed by treatment with the diazonium compound to introduce alkyne terminated groups onto the graphene surface (G-Alk). Subsequently, a click chemistry reaction was performed to covalently bond the graphene platform to CDs. The success of the synthetic route was confirmed *via* TGA, SEM, Raman spectroscopy, and XPS analysis. The working electrode (WE) surface of the SPCE was modified with GCD dispersion *via* drop-casting, and no “coffee-ring” effect was observed, giving a robust electroanalytical determination method. The electrochemical output of the modified electrode (SPCE/GCD) was studied through CV, using [Fe(CN)_6_]^3−^ as the redox probe, and compared to the ones of the bare SPCE. The findings highlight an improvement in the peak current response (by 30%) of SPCE/GCD.

SPCE/GCD was modified with the FcCAR unit (SPCE/GCD/FcCAR), and the formation of the supramolecular complex was confirmed through XPS.

Additionally, the sensing properties of SPCE/GCD/FcCAR towards Mn(ii) were studied using CV and DPV. The latter technique was employed to calculate the LOD and LOQ in the metal concentration range of 0.17 ≤ [Mn(ii)]/nmol L^−1^ ≤ 48. The LOD and LOQ values were found to be 0.69 nmol L^−1^ and 2.3 nmol L^−1^, respectively, with a sensitivity of 0.20 μA nmol^−1^ L^−1^. The LOD value obtained is one of the lowest reported values in the literature.

The present results clearly demonstrate the good analytical performance of SPCE/GCD/FcCAR, making it a promising candidate for Mn(ii) electrochemical detection in aqueous solutions. Further investigations will focus on the development and optimization of the analytical method in natural water samples.

## Author contributions

Conceptualization: G. N., C. A., C. F., and A. P. Funding acquisition: G. N., M. C., and A. P. Investigation: C. A., G. N., M. A., M. C., P. G. M., E. F., C. C., and O. G. Formal analysis: C. A., M. C., P. G. M., E. F., and C. C. Supervision: C. F. and A. P. Writing – original draft: C. F. and G. N. Writing – review and editing: all authors.

## Conflicts of interest

There are no conflicts to declare.

## Supplementary Material

RA-015-D5RA03016A-s001

## Data Availability

The data supporting this article have been included as part of the ESI.†
