# Supplementary material for: Engineered carbon electrode with graphene-cyclodextrin/ferrocenyl-carnosine nanoassembly for Mn(ii) detection
Source: RSC Adv. 2025 Jul 8;15(29):23596–604. doi: 10.1039/d5ra03016a (PMC12235380; doi:10.1039/d5ra03016a)
Supplement: RA-015-D5RA03016A-s001 [file RA-015-D5RA03016A-s001.pdf]

Supplementary Information (SI) for RSC Advances.
This journal is © The Royal Society of Chemistry 2025

*Supporting Information*

# Engineered carbon-electrode with graphene-cyclodextrin/ ferrocenyl-carnosine nanoassembly for Mn(II) detection

Chiara Abate [a], Giulia Neri [a]*, Marco Abbate [a], Massimiliano Cordaro [a], Placido Giuseppe Mineo [b], Enza Fazio [c], Carmelo Corsaro [c], Ottavia Giuffrè [a], Claudia Foti [a]*, and Anna Piperno [a]

[a]*Department of Chemical, Biological, Pharmaceutical, and Environmental Sciences, University of Messina, 31 Viale F. Stagno d'Alcontres, 98166 Messina, Italy;*

[b]*Department of Chemical Sciences, University of Catania, Catania 95125, Italy;*

[c]*Department of Mathematical and Computer Sciences, Physics Science and Earth Science, University of Messina, 31 Viale F. Stagno d'Alcontres, 98166 Messina, Italy.*

## SUMMARY

**Figure S1** 2

**Figure S2** 3

**Figure S3** 4

**Figure S4** 4

**Figure S5** 5

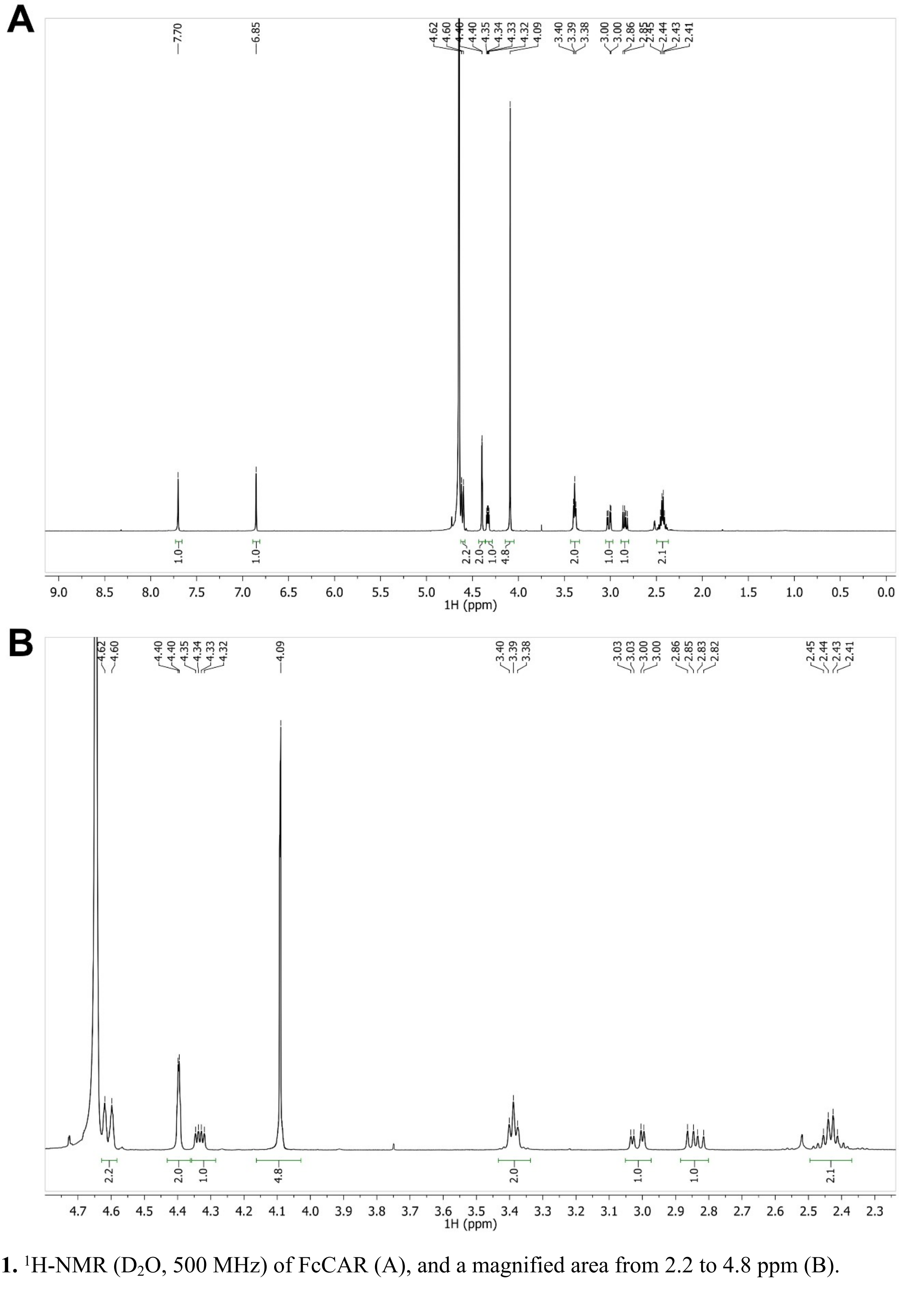


**Fig. S1.** $^1$H-NMR ($D_2O$, 500 MHz) of FcCAR (A), and a magnified area from 2.2 to 4.8 ppm (B).

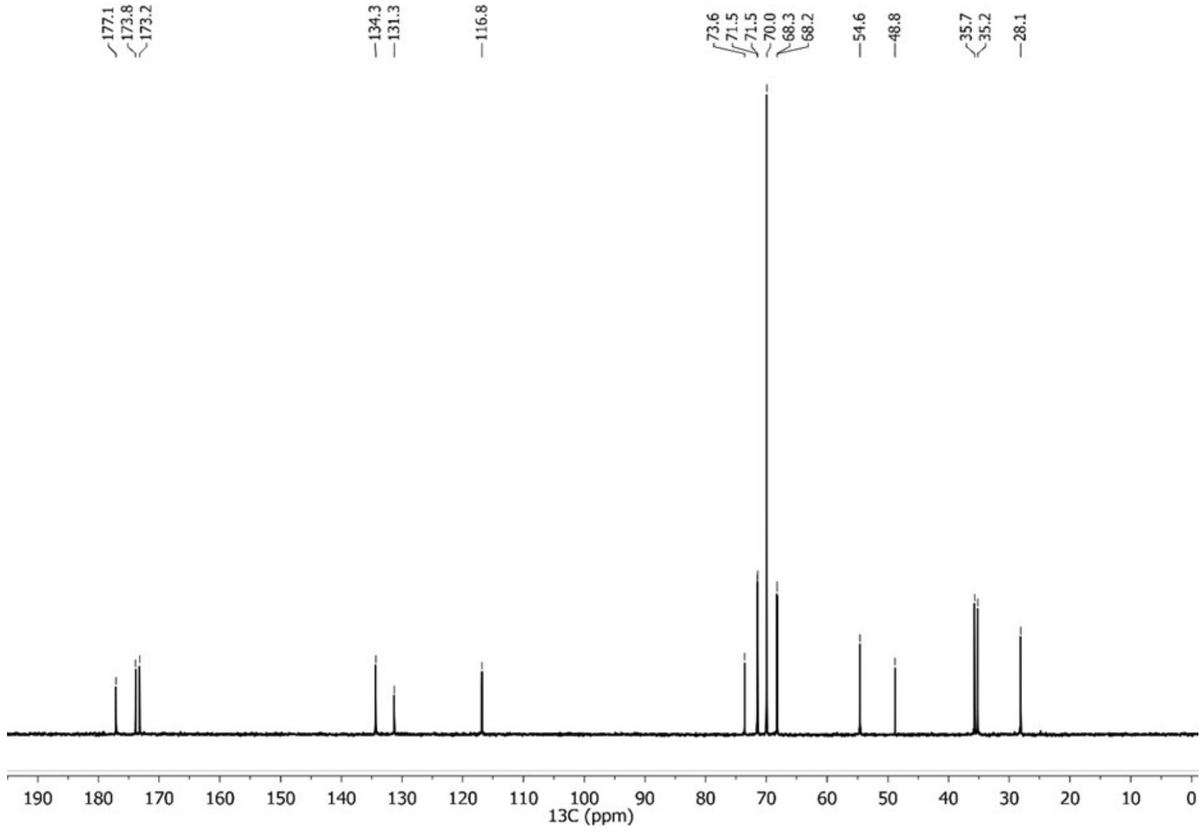


**Fig. S2.** $^{13}C$-NMR ($D_2O$, 125 MHz) of FcCAR.

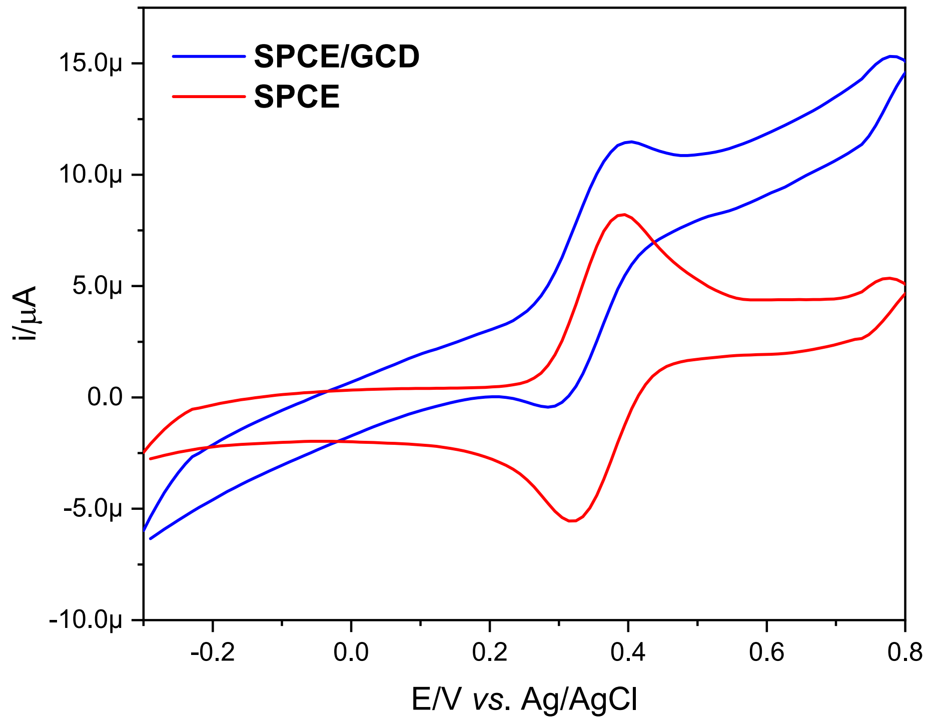


**Fig. S3.** CV response (at 0.1 V s$^{-1}$) of FcCAR (0.5 mmol L$^{-1}$) in KCl (0.1 mol L$^{-1}$) on SPCE/GCD (blue line) and SPCE (red line).

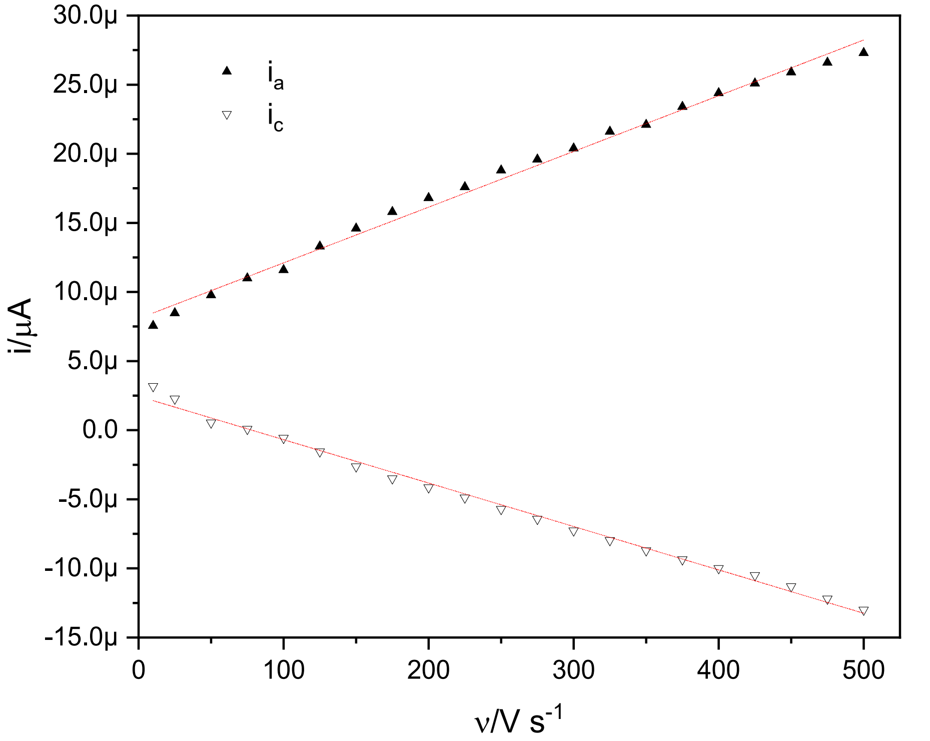


**Fig. S4.** Dependence of anodic ($i_a$) and cathodic ($i_c$) peak currents on the scan rate (ν) for FcCAR (0.5 mmol L$^{-1}$) in KCl (0.1 mol L$^{-1}$) on SPCE/GCD.

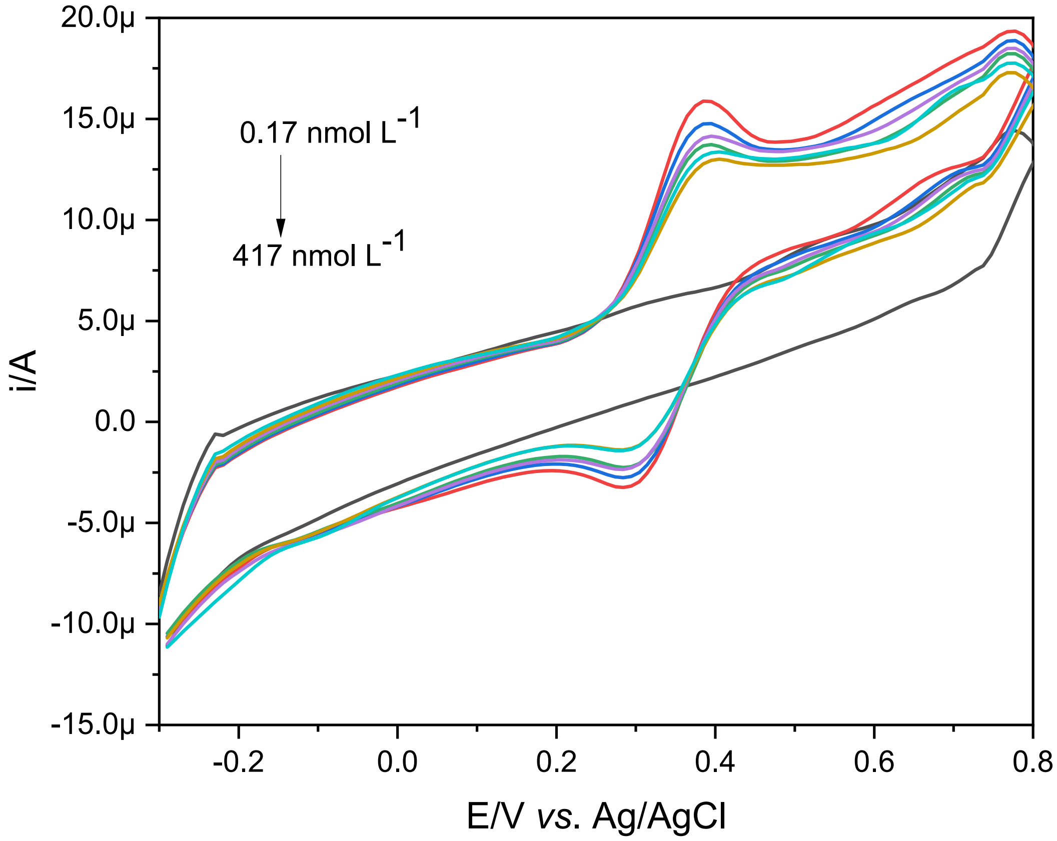


**Fig. S5.** CVs of FcCAR (0.5 mmol $L^{-1}$) at different Mn(II) concentration, compared with the CV of the only Mn(II) (0.8 nmol $L^{-1}$, grey), in KCl (0.1 mol $L^{-1}$) on SPCE/GCD.
